# Massive bleeding and perforation due to post-colectomy pan-enteritis with a significant response to biologic in a patient with ulcerative colitis: a case report

**DOI:** 10.1186/s40792-024-02003-8

**Published:** 2024-08-28

**Authors:** Kenichiro Toritani, Hideaki Kimura, Manabu Maebashi, Kazuki Kurimura, Serina Haruyama, Yoshinori Nakamori, Mao Matsubayashi, Reiko Kunisaki, Reiko Tanaka, Satoshi Fujii, Itaru Endo

**Affiliations:** 1 Inflammatory Bowel Disease Center, Yokohama City University Medical Center, 4-57, Urafune-cho, Minami-ku, Yokohama, 232-0024 Japan; 2 Department of Pathology, Yokohama City University Medical Center, Yokohama, Japan; 3 Department of Molecular Pathology, Yokohama City University Graduate School of Medicine, Yokohama, Japan; 4 Department of Pathology, Yokohama City University Hospital, Yokohama, Japan; 5 Department of Gastroenterological Surgery, Yokohama City University Graduate School of Medicine, Yokohama, Japan

**Keywords:** Ulcerative colitis, Post-colectomy pan-enteritis, Infliximab, Ulcerative colitis-related severe enteritis

## Abstract

**Background:**

Post-colectomy pan-enteritis in ulcerative colitis (UC) is very rare, but it is often severe and fatal. We present a case of massive bleeding and perforation due to post-colectomy pan-enteritis, which showed a significant response to biologics in a UC patient.

**Case presentation:**

A 30-year-old woman with a 5-month history of pancolitis UC underwent subtotal colectomy with ileostomy and mucosal fistula for refractory UC. She was diagnosed with small bowel obstruction on postoperative day (POD) 8 and bowel bleeding was observed on POD18. Reoperation was performed for bowel obstruction and bleeding on POD20. Intraoperatively, adhesive small bowel obstruction in the ileum and multiple erosions and ulcers with perforation were observed throughout the small bowel. We diagnosed post-colectomy pan-enteritis, and jejunostomy, lavage, adhesiolysis, and a simple closure of the perforated ileum were performed. High-dose steroid therapy for pan-enteritis was administered immediately after reoperation, and infliximab was administered because of worsening bleeding on day 3 after reoperation. Bleeding decreased one day after biologic administration and bleeding completely disappeared on day 10 after biologic administration. Specimens obtained from the terminal ileum at colectomy showed a normal ileum without inflammation and villus atrophy, while specimens from the perforated ileum showed congestion, villous atrophy, epithelial erosion, and mononuclear cell infiltration. No cryptitis, crypt distortion, or basal plasmacytosis (common characteristics in UC) were observed in either specimen.

**Conclusion:**

An early diagnosis and intervention are important for post-colectomy pan-enteritis, and infliximab may be effective. Post-colectomy pan-enteritis with a multiple ulcer phenotype has different histological characteristics from UC and may have a different pathogenesis.

## Background

Ulcerative colitis (UC) is typically characterized by superficial diffuse inflammation mainly confined to the rectum and colon, with rare involvement of the small bowel except pouchitis, pre-pouch ileitis, and backwash ileitis [1, 2]. Post-colectomy pan-enteritis is an exceedingly rare complication involving small bowel inflammation in patients with UC, often progressing severely and leading to fatality. Its pathogenesis remains unknown and no treatment has been established [3]. We herein present a case of post-colectomy pan-enteritis with massive bleeding and perforation in patient with UC, which was successfully treated with a biologic agent.

## Case presentation

**Fig. 1:**
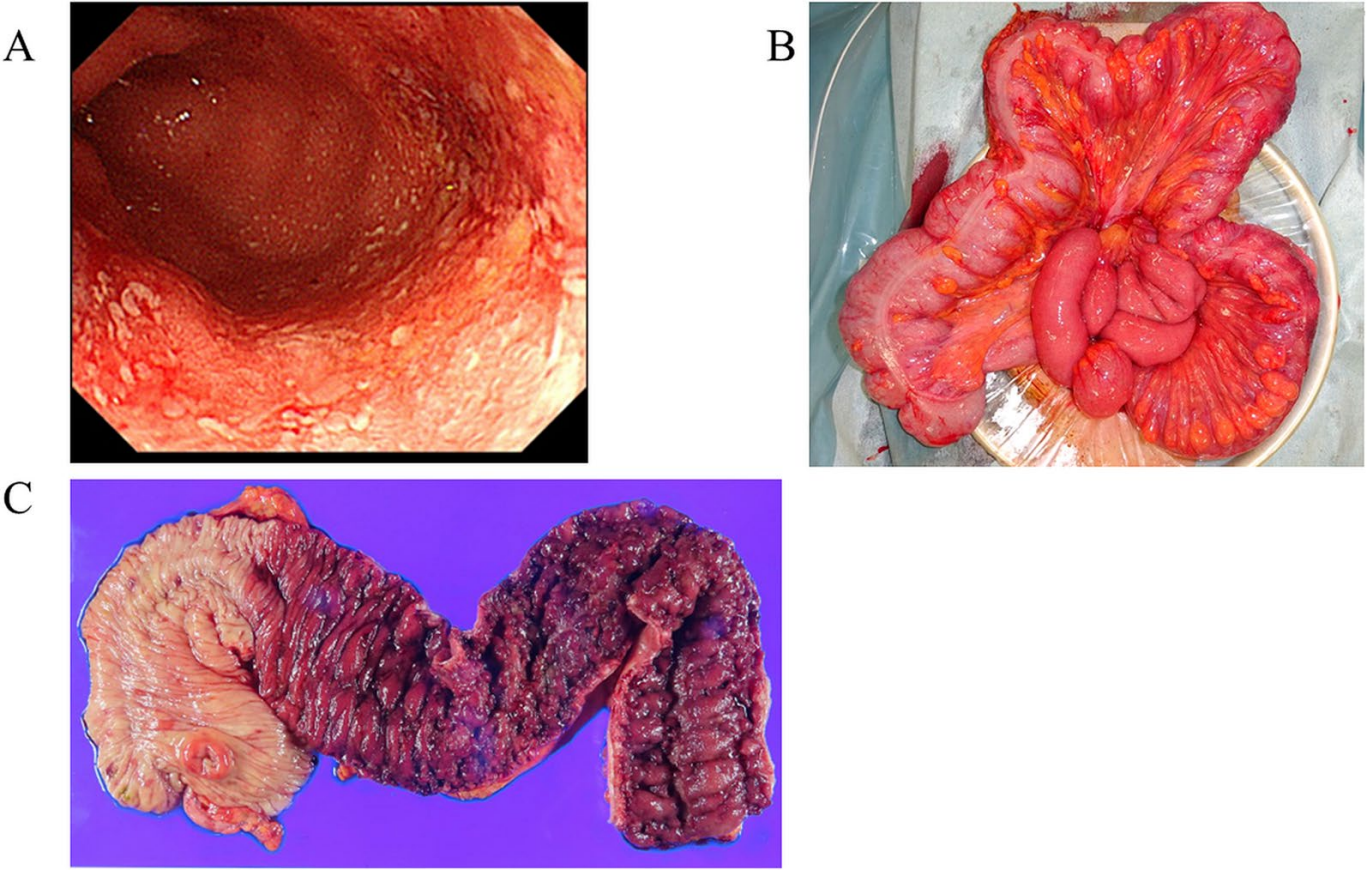
Preoperative and intraoperative findings in colectomy. **A** Preoperative colonoscopy showed continuous diffuse granularity, ulceration, and loss of vascular pattern from the rectum to the transverse colon. **B** Intraoperatively, new vascular growth on the serosal surface from the rectum to the transverse colon was observed. **C** The resection specimen showed pancolitis compatible with UC, and a demarcation line existed at the transverse colon

**Fig. 2:**
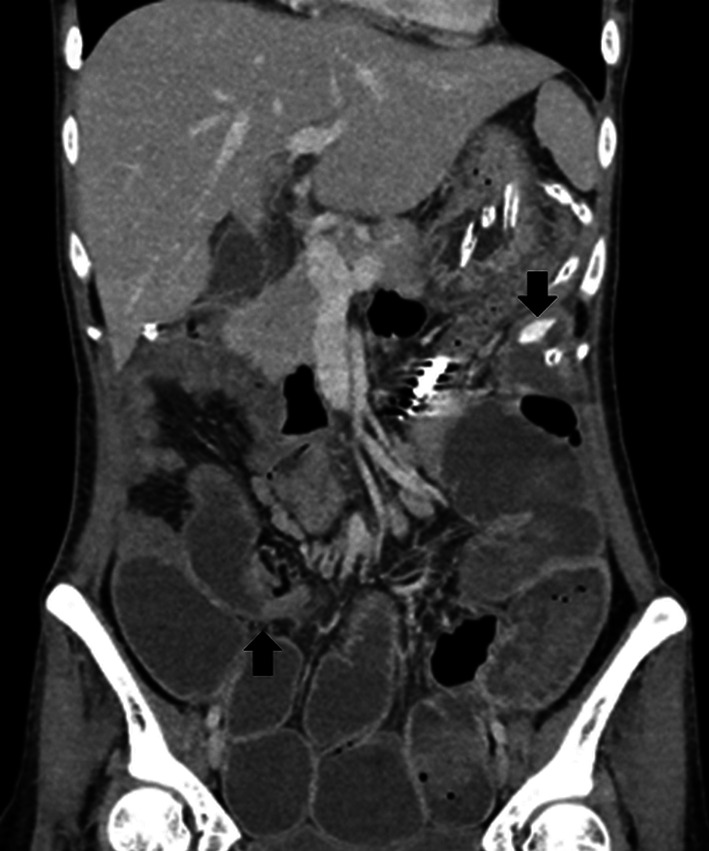
Computed tomography image taken before reoperation. Computed tomography image before reoperation showed extravascular leakage (upper arrow) and caliber change (lower arrow)

**Fig. 3:**
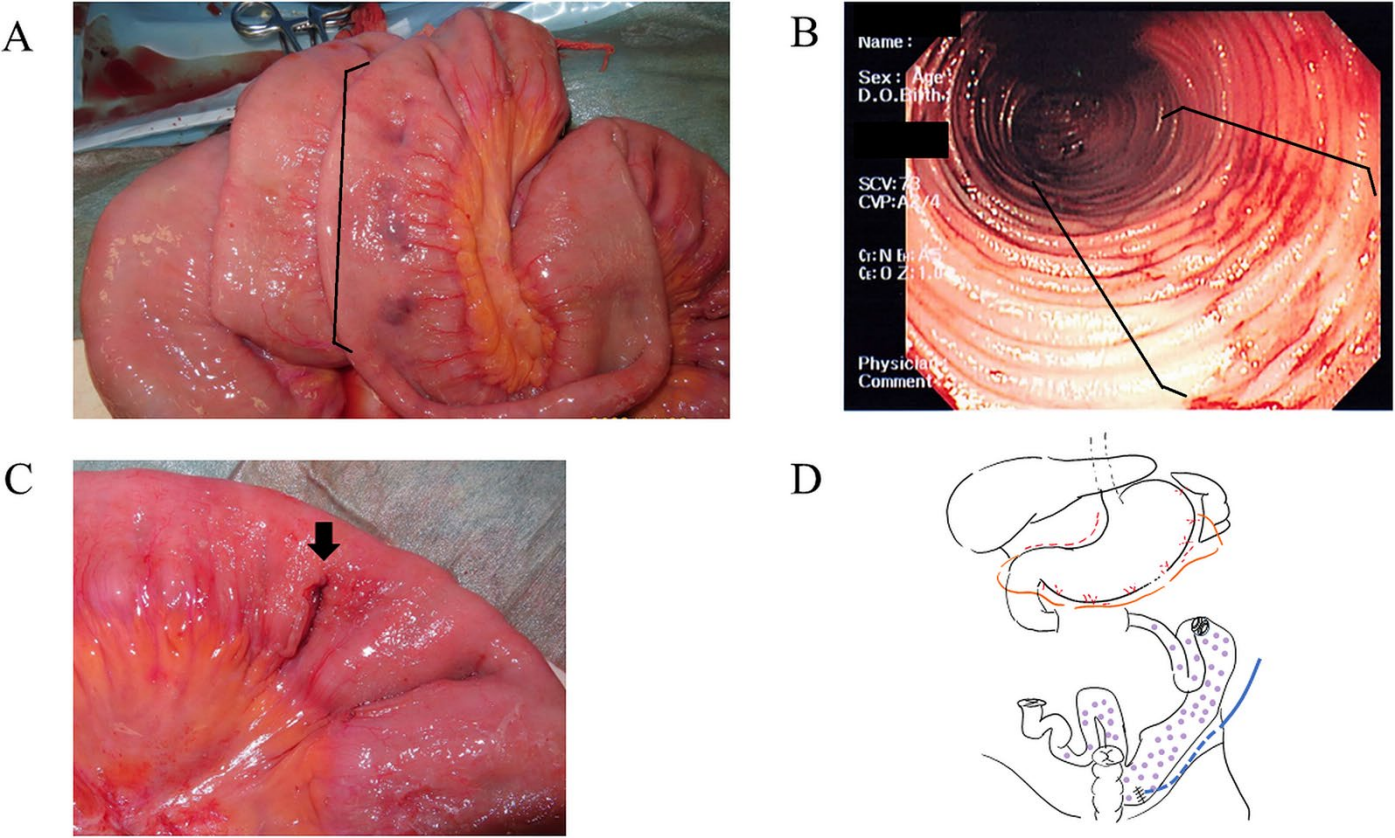
Intraoperative findings in reoperation.** A** Punched-out ulcers were observed in the entire small bowel from the Treitz ligament. **B** Intraoperative endoscopy revealed multiple erosions and ulcers in the small intestine. **C** The ileum was perforated at 220 cm from the Treitz ligament. **D** Jejunostomy, lavage, adhesiolysis, and a simple closure of the perforated ileum were performed

**Fig. 4:**
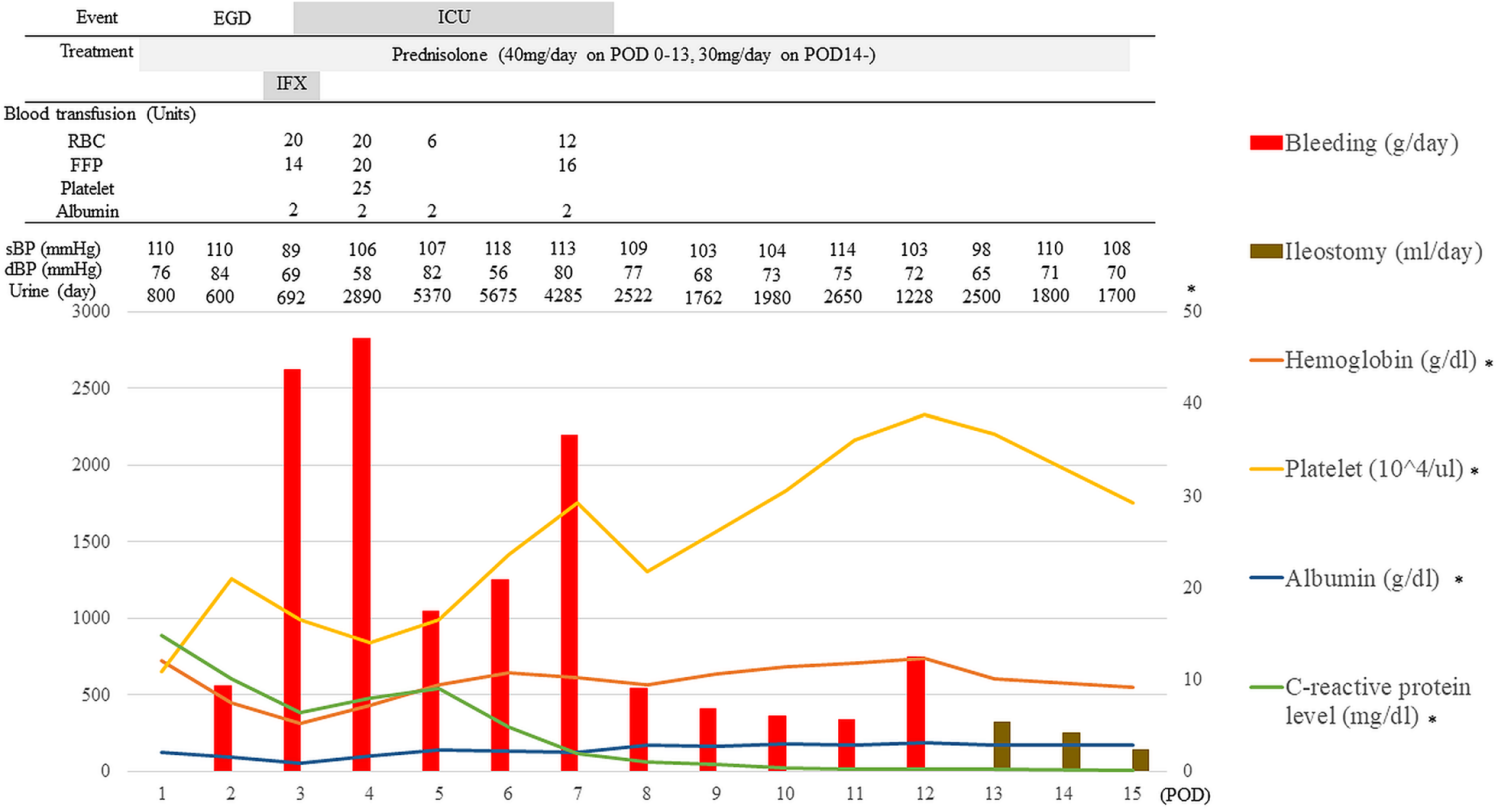
Clinical course after reoperation. Total bleeding was 12898 ml, and blood transfusion with 58 units of red blood cell, 40 units of fresh frozen plasma, and 25 units of platelet were required after reoperation. *EGD* esophagogastroduodenoscopy, *ICU* intensive care unit, *POD* postoperative day, *IFX* infliximab, *RBC* red blood cell, *FFP* fresh frozen plasma, *sBP* systolic blood pressure, *dBP* diastolic blood pressure

A 30-year-old woman with a 5-month history of pancolitis UC presented with an exacerbation of UC and was admitted to a nearby hospital. She had previously been treated with oral 5-aminosalicylic acid (5ASA) medication. She had no significant medical or family history. Oral steroid therapy with prednisone (20 mg/day) was initiated, and she was transferred to our hospital because of an exacerbation of UC (Fig. 1A) on the 14th day of hospitalization. The patient was treated with high-dose intravenous steroid therapy consisting of prednisolone (50 mg/day) and a calcineurin inhibitor. Subtotal colectomy with ileostomy and a mucosal fistula was performed because of refractory UC 5 days after the patient was transferred to our hospital, and neovascular growth on the serosal surface was observed from the rectum to the transverse colon (Fig. 1B). The resected specimen showed pancolitis compatible with UC, and a demarcation line existed in the transverse colon without backwash ileitis (Fig. 1C). On postoperative day (POD) 8, vomiting was noticed and diagnosed as small bowel obstruction by computed tomography (CT). An ileus tube was inserted on POD12. On POD15, fever was observed, and antibiotics were administered for *Enterococcus faecium* sepsis. On POD18, bleeding from the stoma and ileus tube was observed. On POD20, the abdominal pain worsened, and a CT scan revealed bowel obstruction and bleeding (Fig. 2), which led to a reoperation. Intraoperatively, 230 cm from the ligament of Treitz, the ileum was found to be narrowed by adhesions to the ileal mesentery, and multiple erosions and ulcers were observed in the entire small bowel from the ligament of Treitz (Fig. 3A, B). Although there was little inflammation of the mucosa between the ulcers, ulcers were mostly observed in the small bowel, 100 cm from the ligament of Treitz, and the ileum was perforated at 220 cm from the ligament of Treitz (Fig. 3C). Jejunostomy, lavage, adhesiolysis, and a simple closure of the perforated ileum were performed (Fig. 3D). The clinical course after the reoperation is shown in Fig. 4. Esophagogastroduodenoscopy (EGD) after reoperation showed no bleeding in the esophagus, stomach, or duodenum. Tests for cytomegalovirus (CMV) antigenemia and glutamate dehydrogenase, and toxin A/B assays for *Clostridioides difficile* infection were negative. Stool cultures showed normal flora. The patient was diagnosed with post-colectomy pan-enteritis and treated with high-dose steroid therapy with prednisolone (40 mg/day) immediately after reoperation, and infliximab (IFX; 5 mg/kg) was administered due to worsening bleeding on the 3rd day after reoperation. She was admitted to the intensive care unit (ICU) for massive blood transfusion due to bleeding. She required blood transfusion with 58 units of red blood cells, 40 units of fresh frozen plasma, and 25 units of platelets in the ICU, and bleeding decreased on the day after the initiation of biologic therapy. On the 5th day of biologic therapy, the blood transfusion was stopped, and the patient left the ICU. Bleeding from the stoma completely disappeared around the 10th day after the initiation of biologic therapy. The jejunostomy was closed 8 weeks after the reoperation, and the patient was discharged home 5 weeks later. At seven months after colectomy, she is receiving in IFX (5 mg/kg/8 weeks) as maintenance therapy. No rectal perforation or bleeding was observed during the course of this treatment.

**Fig. 5:**
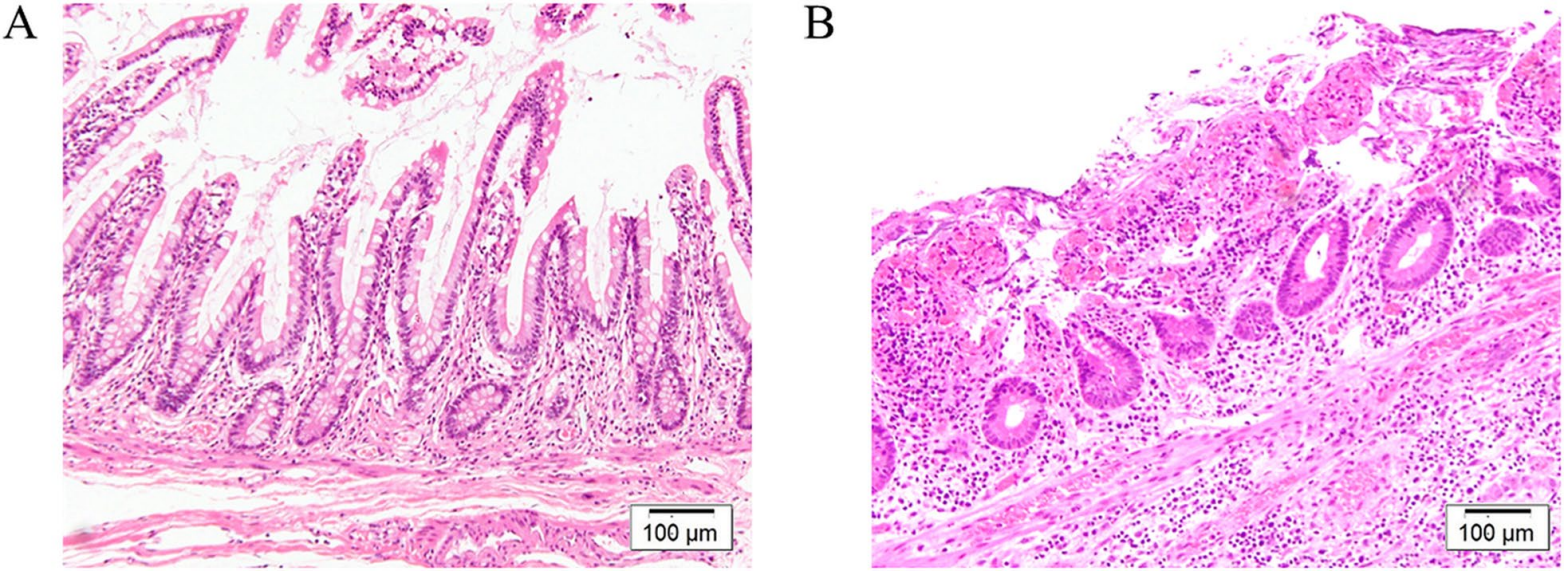
Histological findings of terminal ileum and perforated ileum. Specimens of ileum. No cytomegalovirus infection, cryptitis, distortion of the crypts, basal plasmacytosis, or granulomas were observed in terminal ileum and perforated ileum. **A** Histological finding in terminal ileum revealed no pathological inflammation or villus atrophy. **B** Histological finding in perforated ileum revealed congestion, villous atrophy, and epithelial erosion with severe mononuclear cells infiltration was observed, but neutrophils and eosinophils were mildly infiltrated

The histological findings of a resected specimen obtained from the terminal ileum at colectomy and a specimen obtained from the perforated ileum at reoperation are shown in Fig. 5. The specimen from the terminal ileum showed a normal ileum without pathological inflammation or villus atrophy (Fig. 5A), whereas the specimens from the perforated ileum showed congestion, villous atrophy, epithelial erosion, and mononuclear cell infiltration, with little infiltration of neutrophils and eosinophils (Fig. 5B). Neither specimen showed cryptitis, crypt distortion, basal plasmacytosis, or granuloma. Immunohistochemical staining for CMV was negative in both specimens.

## Discussion 

Post-colectomy pan-enteritis was first described by Mayo et al. in 1963 [4]. It only occurs in patients with UC and is often severe and fatal [5]. Severe post-colectomy pan-enteritis is defined as UC-related severe enteritis (UCRSE), with a frequency of 0.8%, often occurring within 1 month of colorectal resection. Symptoms include massive bleeding, high-output stoma, and perforation, in that order [3]. The present patient was diagnosed with UCRSE after colorectal resection, with massive bleeding from the stoma and intraoperative findings of perforation.

The phenotype of post-colectomy pan-enteritis is not uniform. Uchino et al. [6] reported a case with a UC-like phenotype, exhibiting endoscopic findings of friable and granular mucosa with erosion and multiple aphthae, and histologic findings of polymorph infiltration, cryptitis, and crypt distortion, similar to UC and pouchitis [7]. In contrast, we report a case with a multiple ulcer phenotype, displaying endoscopic findings of multiple erosions and ulcers with a normal intervening mucosa. The histological examination of the perforated ileum revealed characteristics of peripheral circulatory failure, including congestion and villus atrophy. Erosion and infiltration of lymphocytes and plasma cells were also observed, but there was little infiltration of neutrophils, which is similar to the findings reported by Annese et al. [8].

Genetic susceptibility and systemic inflammation may be important factors in UCRSE [3]. The pathogenesis of post-colectomy pan-enteritis with a UC-like phenotype may also be associated with UC or pouchitis; however, that with a multiple ulcer phenotype may not be associated with UC or pouchitis because the endoscopic and histological findings are different. The pathogenesis of post-colectomy pan-enteritis with a multiple ulcer phenotype may be associated with microvasculitis or microthrombosis, as peripheral circulatory disturbances have been observed. The clinical manifestations and endoscopic findings of CMV ileitis are quite similar those of post-colectomy pan-enteritis with a multiple ulcer phenotype [9]. Some cases of post-colectomy pan-enteritis with a multiple ulcer phenotype may be caused by CMV infection because UCRSE is often complicated by CMV enteritis [3.6]. However, CMV infection is not always necessary for post-colectomy pan-enteritis with a multiple ulcer phenotype because CMV antigenemia and immunohistochemical stain for CMV was negative in this case.

Treatment to control systemic inflammation, such as corticosteroids, immunomodulators and immunosuppressor drugs, is effective in some cases of UCRSE [3, 10, 11]. In UCRSE with bleeding, the effective rate of corticosteroids is 47.1% and that of IFX is 70.0% in retrospective multicenter study [3]. IFX is effective in several cases of steroid resistance [5, 6], and the same was true in this case. However, because of the immunosuppressive effects of infliximab, careful attention should be paid to infections, as deaths from pneumonia have been reported after IFX administration for UCRSE [6]. Even in UCRSE with CMV enteritis or *Clostridioides difficile* enteritis, antivirus and antibiotics drug are often not effective [3, 5].

Massive resection of the small bowel for bleeding and infection control was not performed at the time of reoperation because of the risk of permanent postoperative short bowel syndrome. Simple closure with lavage and jejunostomy was performed, and bleeding was controlled with massive blood transfusion. Early biologic administration and close observation to not miss the opportunity for surgical intervention may be important for post-colectomy pan-enteritis because UCRSE is associated with a high rate of mortality, with hemorrhage, infection, and disseminated intravascular coagulation being the main causes of death [3.8].

## Conclusions

An early diagnosis and intervention are important for post-colectomy pan-enteritis, and infliximab may be effective in these cases. Post-colectomy pan-enteritis with a multiple ulcer phenotype has different histologic characteristics and it may have a different pathogenesis from UC and pouchitis.

## Data Availability

All data generated or analyzed during this study are included in the published article.

## Abbreviations

UC: Ulcerative colitis
POD: Postoperative day
CT: Computed tomography
5ASA: 5-Aminosalicylic acid
EGD: Esophagogastroduodenoscopy
ICU: Intensive care unit
CMV: Cytomegalovirus
UCRSE: Ulcerative colitis-related severe enteritis
sBP: Systolic blood pressure
dBP: Diastolic blood pressure
